# Finsler Geometry Modeling of Phase Separation in Multi-Component Membranes

**DOI:** 10.3390/polym8080284

**Published:** 2016-08-04

**Authors:** Satoshi Usui, Hiroshi Koibuchi

**Affiliations:** Department of Mechanical and Systems Engineering, National Institute of Technology, Ibaraki College, Nakane 866, Hitachinaka, Ibaraki 312-8508, Japan; ac14102@gm.ibaraki-ct.ac.jp

**Keywords:** biological membranes, multi-component, phase separation, liquid-ordered, liquid-disordered, line tension, Monte Carlo, surface model, Finsler geometry

## Abstract

A Finsler geometric surface model is studied as a coarse-grained model for membranes of three components, such as zwitterionic phospholipid (DOPC), lipid (DPPC) and an organic molecule (cholesterol). To understand the phase separation of liquid-ordered (DPPC rich) $L_{o}$ and liquid-disordered (DOPC rich) $L_{d}$, we introduce a binary variable σ(=±1) into the triangulated surface model. We numerically determine that two circular and stripe domains appear on the surface. The dependence of the morphological change on the area fraction of $L_{o}$ is consistent with existing experimental results. This provides us with a clear understanding of the origin of the line tension energy, which has been used to understand these morphological changes in three-component membranes. In addition to these two circular and stripe domains, a raft-like domain and budding domain are also observed, and the several corresponding phase diagrams are obtained.

## 1. Introduction

Membranes of multiple components, such as 1,2-dioleoyl-sn-glycero-3-phosphocholine (DOPC), dipalmitoylphosphatidylcholine (DPPC) and cholesterol, are receiving widespread attention because of their applications in many fields of science and technology, and numerous studies on the morphological changes have been conducted [1,2,3,4,5,6]. In these membranes, morphological changes are induced by a phase separation. Indeed, the phase separation causes domain formation and domain pattern transition between the liquid-ordered ($L_{o}$) and the liquid-disordered ($L_{d}$) phases. This domain pattern transition accompanies the morphological changes, such as the two circular domains, the stripe domain, the raft domain and the so-called budding domain [5,6]. The multiplicity of components, as in a glass transition [7], is essential for such a variety of morphologies. To date, these morphologies have been studied on the basis of the line tension energy [3,4] in the context of the Helfrich–Polyakov (HP) model for membranes [8,9]. The line tension energy is defined on the domain boundary and has an important role in the morphological changes [3,4].

However, the origin of the line tension energy is not well understood. In fact, it is unclear what type of internal structure is connected to the line tension energy until now. The problem that should be asked is where the line tension energy originates. Therefore, in this paper, we clarify and discuss the microscopic origin of the line tension energy.

To understand the origin of the line tension energy, we introduce a new degree of freedom σ(=±1) to represent the $L_{o}$ and $L_{d}$ phases. The Ising model Hamiltonian, which we call aggregation energy, for the variable *σ* is included in the general HP model Hamiltonian, where the “general” HP model refers to the HP model with a nontrivial surface metric $g_{ab}\left(\neq \delta_{ab}\right)$. Note that the general HP model can be discretized on triangulated surfaces and becomes well defined only when it is treated in the context of Finsler geometry [10,11,12]. Moreover, note that our strategy towards the multi-component membrane in this paper is a coarse-graining of the detailed information on the chemical structures of DOPC, DPPC and cholesterol and on the interaction between them with the help of the variable *σ* and the HP surface model. In addition, from the viewpoint of modeling, it is very natural to extend the HP model to the general HP model for explaining the morphological changes in multi-component membranes. Indeed, the HP model is considered as a straightforward extension of the linear chain model for polymers [13].

The remainder of this paper is organized as follows. In SubSection 2.1, we introduce the continuous Hamiltonian, which is identical to the Polyakov Hamiltonian [8]. In SubSection 2.2, we introduce the two-component surface model, which is defined by including the aggregation energy in the Hamiltonian of the FG surface model. The aggregation energy is defined by the variable *σ*, which is introduced to label the triangles with $L_{o}$ and $L_{d}$. The Monte Carlo (MC) technique is briefly discussed in Section 3, and the MC results are presented in Section 4. Finally, we summarize the results in Section 5. In Appendix A, we describe the technical details of the FG modeling. In Appendix A.1, the discretization of the continuous model introduced in SubSection 2.1 is described, and a discrete model is obtained. From this discrete model, we obtain the model for two-component membranes by imposing a constraint on the metric function. In Appendix A.2, we show that the models constructed in Appendix A.1 are ill defined in the conventional modeling and that the models become well defined only in the context of Finsler geometry modeling.

## 2. Two-Component Surface Model

### 2.1. Continuous Surface Model

We begin with a continuous surface model, which is defined by the Polyakov Hamiltonian or the Gaussian energy $S_{1}$ for membranes and the bending energy $S_{2}$ with a metric g(x), where $x=(x_{1},x_{2})$ is the local coordinate of the two-dimensional parameter space *M* [14]. Both of the energies are defined by the surface position $r(\in {ℜ}^{3})$, such that:

(1) $$\begin{array}{c} \begin{array}{cc} S_{1} & =\int \sqrt{g}d^{2}xg^{ab}\frac{\partial r}{\partial x_{a}}\cdot \frac{\partial r}{\partial x_{b}},\\ S_{2} & =\frac{1}{2}\int \sqrt{g}d^{2}xg^{ab}\frac{\partial n}{\partial x_{a}}\cdot \frac{\partial n}{\partial x_{b}}, \end{array} \end{array}$$

where *g* is the determinant of the 2 × 2 matrix $g_{ab}$ of the metric function and $g^{ab}$ is its inverse [14]. The symbol n denotes a unit normal vector of the surface. Both $S_{1}$ and $S_{2}$ are conformally invariant. The conformal invariance is a property in which a scale change $g_{ab}\left(x\right)\to f\left(x\right)g_{ab}\left(x\right)$ is not reflected in both $S_{1}$ and $S_{2}$ for any positive function *f*. Two metrics *g* and $g^{\prime }$ are called “conformally equivalent” if a function f(x) exists, such that $g_{ab}^{\prime }=f\left(x\right)g_{ab}$ [14].

For the case where $g_{ab}\left(x\right)$ is given by the Euclidean metric $g_{ab}=\delta_{ab}$ (or the induced metric $g_{ab}=\partial_{a}r\cdot \partial_{b}r$), the surface shape r in ${ℜ}^{3}$ is treated from the perspective of statistical mechanics. These are the HP model [8,9] corresponding to polymerized membranes, and the HP model and the Landau–Ginzburg model [15] have been thoroughly investigated [16,17,18,19,20,21,22].

### 2.2. Discrete Model

**Figure A1:**
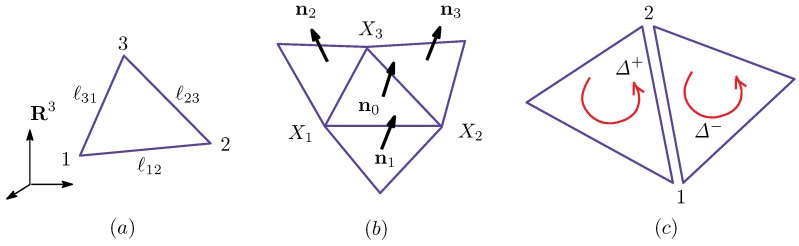
(**a**) A triangle *Δ* included in a triangulated sphere in ${ℜ}^{3}$; (**b**) the three nearest neighbor triangles of *Δ* and the unit normal vectors $n_{0}$, $n_{1}$, $n_{2}$ and $n_{3}$; and (**c**) the triangle orientation that defines the direction-dependent bond potential $\gamma_{12}\ell_{12}^{2}$ and $\gamma_{21}\ell_{21}^{2}$ of the bond 12, where $\ell_{12}=\ell_{21}$.

First, in this subsection, let us introduce a new degree of freedom *σ*, which has only two different values (σ=±1), on the triangulated lattice (see Figure A1 in Appendix A). We assume that the variable $\sigma_{i}$ is defined on the triangle $\Delta_{i}$, and moreover, the values of $\sigma_{i}$ correspond to two different phases, namely the liquid-ordered ($L_{o}$) and the liquid-disordered ($L_{d}$) phases, such that:

(2) $$\begin{array}{c} \sigma \left(\Delta \right)=\left\{\begin{array}{cc} 1 & \;\;\left(\Delta \in L_{o}\right)\\ -1 & \;\;\left(\Delta \in L_{d}\right). \end{array}\right. \end{array}$$

This definition of *σ* implies that every triangle is labeled by the value of *σ*, and therefore, *σ* represents the phase (or domain) to which the triangle *Δ* belongs.

We now introduce a discrete Hamiltonian for multi-component membranes. The technical details of the discretization of the continuous Hamiltonian $S_{1}$ and $S_{2}$ introduced in SubSection 2.1 are described in Appendix A.1, and the discrete expressions for $S_{1}$ and $S_{2}$ are given in Equation (A8) in Appendix A.1. Using these $S_{1}$ and $S_{2}$, we have the total Hamiltonian *S*, such that:

(3) $$\begin{array}{c} S\left(r,\sigma \right)=\lambda S_{0}+S_{1}+\kappa S_{2},\\ S_{0}\left(\sigma \right)=\sum_{ij}\left(1-\sigma_{i}\cdot \sigma_{j}\right),\\ S_{1}\left(r,\sigma \right)=\sum_{ij}\gamma_{ij}\left(\sigma \right)\ell_{ij}^{2},\\ S_{2}\left(r,\sigma \right)=\sum_{ij}\kappa_{ij}\left(\sigma \right)\left(1-n_{i}\cdot n_{j}\right), \end{array}$$

where $S\left(r,\sigma \right)$ denotes that the Hamiltonian depends on the variables $r(\in {ℜ}^{3})$ and *σ*. The three-dimensional vector r denotes the vertex position of the triangulated lattice. The energy $\lambda S_{0}$ is called the aggregation energy. When λ→0, the variable *σ* becomes random, and this random configuration simply corresponds to the coexistence phase, where $L_{o}$ and $L_{d}$ are not separated. Conversely, when *λ* becomes sufficiently large, two neighboring *σ*’s have the same *σ*, and this configuration corresponds the phases where $L_{o}$ and $L_{d}$ are separated. As described above, the second and third terms $S_{1}$ and $S_{2}$ in *S* are the discrete Hamiltonians corresponding to the continuous ones introduced in Section 2.1. The coefficient *κ* of $S_{2}$ is the bending rigidity and has units of $\left[1/k_{B}T\right]$, where $k_{B}$ and *T* are the Boltzmann constant and the temperature, respectively. In this paper, we assume that $k_{B}T=1$. The symbol $n_{i}$ in $S_{2}$ expresses a unit normal vector of the triangle *i*. The symbols $\gamma_{ij}\left(\sigma \right)$ and $\kappa_{ij}\left(\sigma \right)$ denote that $\gamma_{ij}$ and $\kappa_{ij}$ depend on the variable *σ*, and this dependence arises from an interaction between *σ* and r. The interaction between *σ* and r is defined by the function *ρ* (see Equation (A1) in Appendix A.1), such that:

(4) $$\begin{array}{c} \rho \left(\Delta \right)=\left\{\begin{array}{cc} c & \;\;\left(\Delta \in L_{o}\Leftrightarrow \sigma \left(\Delta \right)=1\right)\\ 1 & \;\;\left(\Delta \in L_{d}\Leftrightarrow \sigma \left(\Delta \right)=-1\right), \end{array}\right. \end{array}$$

where *c* is a parameter that should be fixed at the beginning of the simulations.

**Figure 1:**
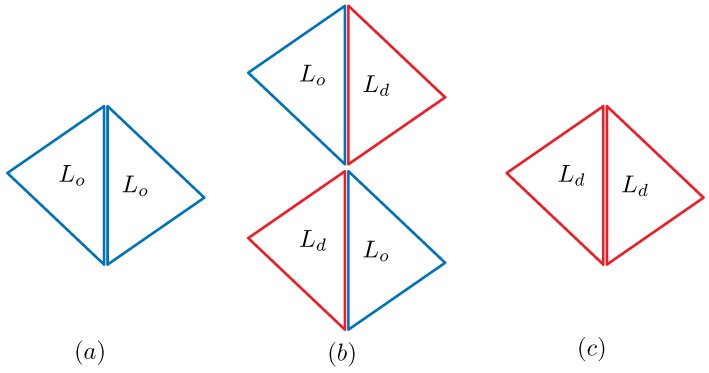
The dependence of $\kappa_{ij}$ and $\gamma_{ij}$ on four possible combinations of $L_{o}$ and $L_{d}$: (**a**) $\kappa_{ij}=\gamma_{ij}=\left(c+c^{-1}\right)/2$ on $\left(L_{o},L_{o}\right)$; (**b**) $\kappa_{ij}=\gamma_{ij}=\left(2+c+c^{-1}\right)/4$ on $\left(L_{o},L_{d}\right)$; and (**c**) $\kappa_{ij}=\gamma_{ij}=1$ on $\left(L_{d},L_{d}\right)$. $\left(L_{o,d},L_{o,d}\right)$ correspond to the bonds represented by the duplicated lines.

From this definition of ρ(Δ) and Equation (A9) in Appendix A.1, we have (see Figure 1):

(5) $$\begin{array}{c} \begin{array}{cc} \gamma_{ij} & =\kappa_{ij}=\frac{c_{i}+c_{j}}{4}=\frac{1}{4}\left(\rho_{i}+\frac{1}{\rho_{i}}+\rho_{j}+\frac{1}{\rho_{j}}\right)\\ & =\left\{\begin{array}{cc} (c+c^{-1})/2 & \;\left[\sigma_{i}=\sigma_{j}=1\;:\;\left(L_{o},L_{o}\right)\right]\\ (2+c+c^{-1})/4 & \;\left[\sigma_{i}\sigma_{j}=-1\;:\;\left(L_{o},L_{d}\right)\right]\\ 1 & \;\left[\sigma_{i}=\sigma_{j}=-1\;:\;\left(L_{d},L_{d}\right)\right]. \end{array}\right. \end{array} \end{array}$$

These expressions represent how the effective surface tension $\gamma_{ij}$ and bending rigidity $\kappa \kappa_{ij}$ depend on the position of the bond ij, which is one of the three domain boundary bonds $\left(L_{o},L_{o}\right)$, $\left(L_{o},L_{d}\right)$ and $\left(L_{d},L_{d}\right)$. The symbol $\left(L_{o},L_{d}\right)$ refers to the bond shared by the two neighboring triangles of the $L_{o}$ and $L_{d}$ phases (see Figure 1). Note that only $\left(L_{o},L_{d}\right)$ corresponds to the bond on the domain boundary, and the other two correspond to the bonds inside the domains $L_{o}$ and $L_{d}$. From the expressions of $\gamma_{ij}$ and $\kappa_{ij}$ in Equation (5), we understand that the dependence of $\gamma_{ij}$ and $\kappa_{ij}$ on the domains and their boundary is automatically determined. Thus, this expression is one of the most interesting outputs of the model in this paper. The values of $\gamma_{ij}$ and $\kappa_{ij}$ depend on the parameter *c*, which is an input parameter.

**Figure 2:**
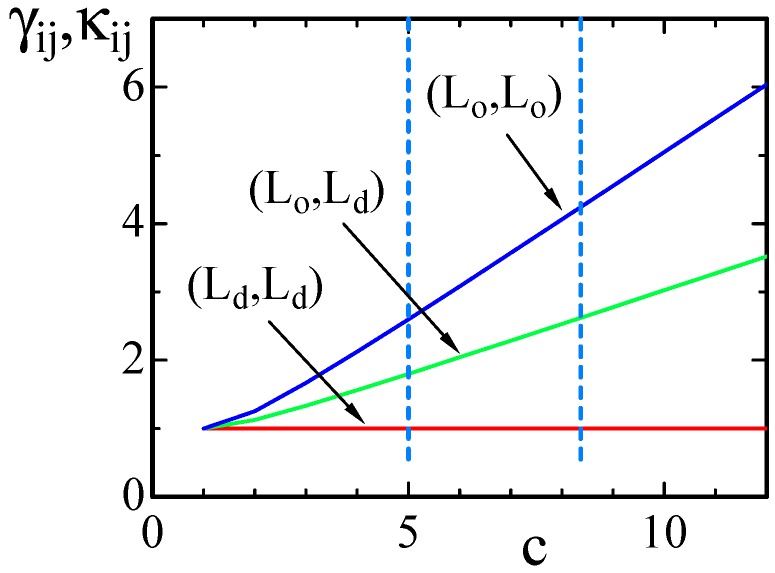
Three different values of $\gamma_{ij}$ and $\kappa_{ij}$ vs. *c*, where $\kappa_{ij}=\gamma_{ij}=\left(c+c^{-1}\right)/2$ on the $\left(L_{o},L_{o}\right)$ boundary, $\kappa_{ij}=\gamma_{ij}=\left(2+c+c^{-1}\right)/4$ on the $\left(L_{o},L_{d}\right)$ boundary and $\kappa_{ij}=\gamma_{ij}=1$ on the $\left(L_{d},L_{d}\right)$ boundary. The dashed lines denote the values of *c* assumed in some of the simulations.

In Figure 2, $\gamma_{ij}\left(=\kappa_{ij}\right)$ for $\left(L_{o},L_{o}\right)$, $\left(L_{o},L_{d}\right)$ and $\left(L_{d},L_{d}\right)$ are plotted as functions of *c* in the region 1≤c. The expressions of $\gamma_{ij}$ and $\kappa_{ij}$ in Equation (5) are symmetric under the exchange c↔1/c, and therefore, we use the value of *c* rather than 1/c to represent $\gamma_{ij}$ and $\kappa_{ij}$. The curve of $\gamma_{ij}\left(=\kappa_{ij}\right)$ against *c* is almost linear except for the region c≃1. The dashed vertical lines in the figure correspond to c=5 and c=8.37, which are assumed in the simulations.

The fluid surface model is defined by the sum over all possible triangulations $\sum_{T}$ in the partition function, such that:

(6) $$\begin{array}{c} Z\left(\lambda ,\kappa \right)=\sum_{T}\int^{\prime }\prod_{i=1}^{N}dr_{i}\exp\left[-S(r,\sigma )\right], \end{array}$$

where the prime in $\int^{\prime }$ denotes that the center of mass of the surface is fixed at the origin of ${ℜ}^{3}$ to protect the surface translation. The dynamical triangulation, denoted by $\sum_{T}$, is performed using the bond flip technique [23,24,25,26,27,28]. Due to this bond flip, the vertices can freely diffuse over the surface, where two neighboring triangles merge and split into two different ones, and the total number of triangles remains unchanged in this process. Therefore, not only the vertices, but also the triangles diffuse over the surface.

Note that the metric variable, or in other words, the function *ρ*, is not summed over (or integrated out) in *Z*; hence, strictly speaking, it is not a dynamical variable. However, the metric variable *ρ* is effectively considered as dynamical in the sense that *ρ* changes its value on the surface due to the diffusion of triangles.

Moreover, note that the aggregation energy $\lambda S_{0}$ simply corresponds to the line tension energy in [3,4]. Indeed, the energy $1-\sigma_{i}\cdot \sigma_{j}$ at the bond ij has a non-zero positive value only when the bond is on the domain boundary between $L_{o}$ and $L_{d}$. More precisely, $S_{0}$ is proportional to the total number of bonds that form the domain boundary because the mean bond length is constant (or non-zero finite) on the boundary.

We comment on the reason why $\lambda S_{0}$ is considered as the line tension energy in more detail. First, the fact that the mean bond length becomes constant is understood from the scale-invariant property of the partition function *Z* in Equation (6). Indeed, we have $\langle S_{1}\rangle /N=3/2$ [29]. It is easy to see that this relation is satisfied: *Z* is independent of the scale change r→αr for arbitrary α∈ℜ, and therefore, we have ${\left.dZ(\alpha )/d\alpha \right|}_{\alpha =1}=0$. Because $Z\left(\alpha \right)=\alpha^{3N-1}\sum_{T}\int^{\prime }\prod_{i=1}^{N}dr_{i}\exp\left[-S(\alpha r)\right]$, $S\left(\alpha r\right)=\lambda S_{0}+\alpha^{2}S_{1}+\kappa S_{2}$, we have $S_{1}/N=1.5$ for sufficiently large *N*. The relation $\langle S_{1}\rangle /N=3/2$ means that $\langle \gamma_{ij}\ell_{ij}^{2}\rangle$ is constant. This implies that $\langle \ell_{ij}^{2}\rangle$, and hence, $\langle \ell_{ij}\rangle$ becomes constant. This constant $\langle \ell_{ij}\rangle$ varies depending on whether the bond ij belongs to $L_{o}$, $L_{d}$ or the boundary between $L_{o}$ and $L_{d}$ because the coefficient $\gamma_{ij}$ varies depending on these domains and the domain boundary, as in Equation (5). For this reason and because $\langle \gamma_{ij}\ell_{ij}^{2}\rangle =\mathrm{constant}$, we understand that the bond length on the boundary between $L_{o}$ and $L_{d}$ becomes well defined (or non-zero finite). Importantly, the mean bond length is expected to be finite, although it fluctuates around the mean value, and the mean value itself varies depending on the domains or the domain boundary. Therefore, $\lambda S_{0}$ is considered to be an extension of the line tension energy because $S_{0}$ is proportional to the length of the phase boundary if the two phases are clearly separated as the domains $L_{o}$ and $L_{d}$ at least.

The remaining problem to be clarified is how the domain boundary is formed on the triangulated surfaces. During experiments, the area fraction of $L_{o}$ (and $L_{d}$) is fixed [3]. Hence, in our model, the total number of triangles $N_{T}^{o}$ for σ=1 (and $N_{T}^{d}$ for σ=−1) is fixed, where the total number of triangles:

(7) $$\begin{array}{c} N_{T}=N_{T}^{o}+N_{T}^{d} \end{array}$$

is also fixed to be constant (because $N_{T}=2N-4$ and the total number *N* of vertices is fixed). The relation between the area fraction of $L_{o}$ and the fraction of $N_{T}^{o}$ will be described in the next section. Another constraint imposed on the triangles in our model is that the value of $\sigma_{i}$ of triangle *i* remains unchanged for all *i* during the simulations. Therefore, the triangles themselves have to diffuse over the surface to form the $L_{o}$ and $L_{d}$ domains. This triangle diffusion is numerically possible on the dynamically-triangulated surfaces, which are called triangulated fluid surfaces, via the Monte Carlo (MC) technique with dynamical triangulation, as described above [23,24,25,26,27,28].

The function ρ(Δ) in Equation (4) characterizes the difference between the phases $L_{o}$ and $L_{d}$ of *Δ*, and these two different phases are labeled by the variable σ(Δ) as in Equation (2). Therefore, the model in this paper is limited to membranes with two-component domains; however, the modeling technique is applicable to membranes with multi-component domains. Here, we comment on how to extend the model to a *n*-component model. To extend the two-component model, we have to define the value of ρ(Δ) for the *n*-component model, such that $\Delta \in L_{i}\left(1\leq i\leq n\right)$ (see Equation (4)), where $\left\{L_{1},L_{2},\cdots ,L_{n}\right\}$ is the set of domains assumed. In this case, the variable σ(Δ) should be *n* components, and therefore, the corresponding energy term $\lambda S_{0}$ in Equation (3) should also be extended. The Hamiltonian of the *n*-states Potts model, for example, can be used for $S_{0}$. Hamiltonians of continuous models, such as the Heisenberg spin model, can also be assumed for $S_{0}$, where the continuous variable σ(Δ) should be connected in one-to-one correspondence with the *n*-component function ρ(Δ) (see Equation (4)). In these *n*-component models, the energy $\lambda S_{0}$ is still expected to play the role of line tension energy between two different domains, because $\lambda S_{0}$ becomes zero (nonzero) if the phases of two neighboring triangles are identical to (different from) each other. The parameters $\kappa_{ij}$ and $\gamma_{ij}$ are given by the same expression in Equation (A9); however, the final expression of these parameters in the *n*-component model are in general different from those in Equation (5) because of the dependence of the parameters on the definition of $\rho_{i}$. Indeed, the parameters $\kappa_{ij}$ and $\gamma_{ij}$ in the *n*-component model will be different from those determined by the single parameter *c* in the two-component model.

## 3. Monte Carlo Technique

The canonical Metropolis technique is used [30,31]. The vertex position r is updated, such that $r^{\prime }=r+\delta r$. The symbol δr denotes a random three-dimensional vector in a sphere of radius *R*. The new position $r^{\prime }$ is accepted with probability Min[1,exp(−δS)], where δS=S(new)−S(old). The radius *R* of the small sphere is fixed such that the acceptance rate for the update of r is approximately equal to 50%.

The triangulation T is updated using the bond flip technique, as described in the previous section [23,24,25,26,27,28]. We use the same technique used in [23,24,25,26,27,28], except for the following constraint. In the bond flip, the two neighboring triangles of the bond change to a new pair of triangles, such that the fraction $\phi_{0}$ of $L_{o}$ (or $L_{d}$) remains unchanged, where $\phi_{0}$ is defined by:

(8) $$\begin{array}{c} \phi_{0}=N_{T}^{o}/N_{T}. \end{array}$$

More precisely, if the two triangles have the same value of *σ* prior to the bond flip, then the new values of *σ* for the new triangles are fixed to be the same as the old one. However, if the values of *σ* are different from each other before the bond flip, then the new values are also fixed randomly to be different. Only through this process is the variable *σ* updated. Due to this update of *σ* through the dynamical triangulation, the function *ρ* changes, and hence, a domain structure of $L_{o}$ (or $L_{d}$) is formed on the surface.

We comment on the relation between the fraction $\phi_{0}$ and the area fraction of $L_{o}$. As described in the previous section, the mean triangle areas $a_{o}$ and $a_{d}$ in the domains $L_{o}$ and $L_{d}$ are constant because of the scale invariance of *Z*. Therefore, the area fraction of $L_{o}$ can be written as $N_{T}^{o}a_{o}/\left(N_{T}^{o}a_{o}+N_{T}^{d}a_{d}\right)$, which is identical to $\phi_{0}=N_{T}^{o}/N_{T}$ if $a_{o}=a_{d}$. However, the area fraction of $L_{o}$ is not always reflected in the fraction $\phi_{0}$ if $a_{o}\neq a_{d}$.

The initial configuration for the simulations is fixed to be the random phase, where the $L_{o}$ (or $L_{d}$) triangles are randomly distributed on the surface under a constant ratio $\phi_{0}$. This random state corresponds to the two-phase coexistence configuration.

A single Monte Carlo sweep (MCS) consists of *N* updates of r and of *N* updates for the bond flips. The total number of MCS that should be performed depends on the parameters; it ranges from approximately $1\times 10^{8}$ to $8\times 10^{8}$. The numbers of MCS for almost all simulations are $2\times 10^{8}∼3\times 10^{8}$. The simulations at the phase boundaries are relatively time consuming in general because the domain structure and, hence, the surface shape change very slowly at these boundaries. The total number of vertices *N* is fixed to N=5762 in this paper.

## 4. Simulation Results

Two types of models, which are denoted as Model 1 and Model 2, are simulated. The Gaussian energy $S_{1}=\sum_{ij}\ell_{ij}^{2}$ of the canonical surface model is assumed for Model 1. From this assumption, the effective surface tension $\gamma_{ij}$ for Model 1 is $\gamma_{ij}=1$. Model 2 is the same as the one introduced in Section 2.2. The Gaussian energy $S_{1}$ and the parameters $\gamma_{ij}$ and $\kappa_{ij}$ for Model 1 and Model 2 are presented in Table 1.

In Model 1, the surface shape is influenced only by $\kappa_{ij}$ because $\gamma_{ij}=1$. In contrast, in Model 2, the coefficient $\gamma_{ij}$ influences the surface size because $S_{1}$ in Equation (3) has the unit of length squares. Indeed, as described in Section 2, from the scale invariance of *Z*, we have $\langle S_{1}\rangle /N=3/2$ [29], and therefore, $\ell_{ij}^{2}$ deviates from the constant expected from this relation if the constraint $\gamma_{ij}=1$ is not imposed on $\gamma_{ij}$. For example, if $\gamma_{ij}$ is large (small), then $\ell_{ij}^{2}$ becomes small (large). Therefore, due to this dependence of $\ell_{ij}^{2}$ on $\gamma_{ij}$, the size of the triangles in Model 2 depends on the domains. By contrast, there is no dependence of $\ell_{ij}^{2}$ on the domains in Model 1, where $\gamma_{ij}=1$ over the entire surface.

The input parameters for the simulations are *λ*, *κ*, *c*, and $\phi_{0}$, where *c* is the value of the function *ρ* in Equation (4) and determines $\gamma_{ij}$ and $\kappa_{ij}$. The parameter $\phi_{0}$ defined by Equation (8) is identical to the area fraction in Model 1, whereas it differs from the area fraction in Model 2 because the triangle area is not uniform in Model 2. More precisely, the mean triangle area in the $L_{o}$ domain is different from that in the $L_{d}$ domain in Model 2. In Table 2, we show the parameters assumed in the simulations. The values of $\kappa_{ij}$ corresponding to the input *c* are listed in Table 3.

### 4.1. Model 1

**Figure 3:**
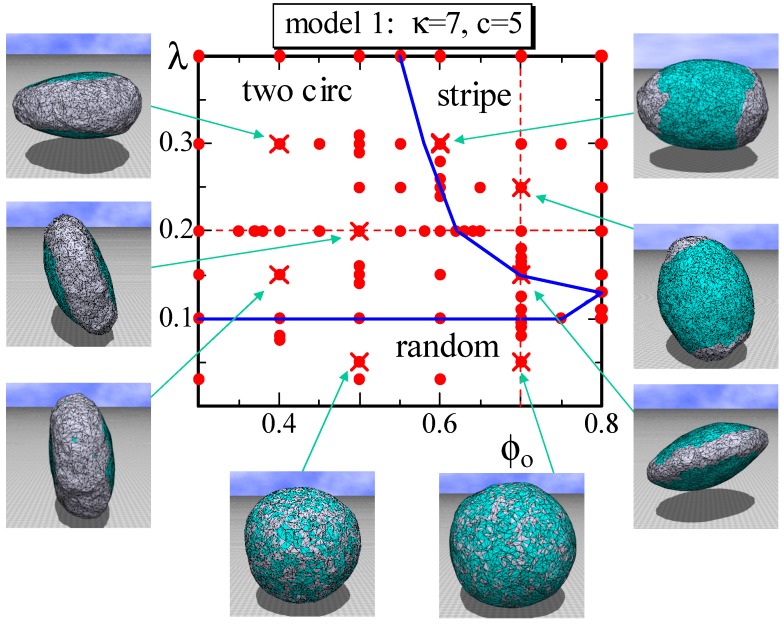
A phase diagram of Model 1 on the $\lambda -\phi_{o}$ plane at κ=7 and c=5 and the snapshots of surfaces obtained at the points indicated by the symbol (×). The solid lines denote the phase boundaries, and the dashed lines denote the positions for the simulations for Figure 4a–c. The solid circles (•) denote the data points of the simulations for the phase boundaries. The two circular domains and the stripe domain correspond to the $L_{o}$ phase, which is DPPC rich. The two separated domains on the surface of the striped domain and the connected domain on the surface of two circular domains correspond to the $L_{d}$ phase, which is DOPC rich.

We first show a phase diagram on the $\lambda -\phi_{o}$ plane in Figure 3. The parameters *κ* and *c* are fixed to κ=7 and c=5, as shown in Table 1, and *λ* is varied in its relatively small region. The dots (•) are the data points where we perform the simulations to construct the phase diagram. We find that the two phases $L_{o}$ and $L_{d}$ are not separated in the region λ<0.1; the domain pattern is random; and the surface is almost spherical, as observed in the snapshots. In contrast, in the region λ>0.1, $L_{o}$ and $L_{d}$ are clearly separated, and the two circular domains and the stripe domain appear. The domain structure depends on the value of $\phi_{0}$, and the corresponding surface morphology appears to be almost discontinuously separated on the phase diagram. We observe that the two circular domains change to the stripe domain as the fraction $\phi_{0}$, which is identical with the area fraction of $L_{o}$, increases for constant *λ*. This result is consistent with the experimental results reported in [3], where the area fraction of $L_{o}$ is changed. The two circular domains and the stripe domain correspond to the $L_{o}$ phase, where $\kappa_{ij}$ is higher than those of both the $L_{d}$ domain and the boundary, as shown in Table 1. For this reason, the $L_{o}$ domain is relatively smooth compared to the $L_{d}$ domain. The ratio $\kappa_{ij}\left(L_{o},L_{o}\right)/\kappa_{ij}\left(L_{d},L_{d}\right)\left(=2.5∼4.3\right)$ assumed in the simulations is almost comparable to the experimental prediction $\kappa_{ij}\left(L_{o},L_{o}\right)/\kappa_{ij}\left(L_{d},L_{d}\right)\left(=1∼4\right)$ [3].

Next, to show the dependence of the surface size on the parameters, we define semi-axis lengths $D_{1}$, $D_{2}$ and $D_{3}$ of the surface such that $D_{1}>D_{2}>D_{3}$ as in Figure 5. $D_{1}$ and $D_{2}$, $D_{3}$ correspond to the major and minor axes, respectively. The surface of the stripe domain corresponds to the so-called prolates, where $D_{1}>D_{2}≃D_{3}$ is expected. It is also expected that $D_{1}≃D_{2}>D_{3}$ in the so-called oblates, which corresponds to the surface shape of the two circular domains. Therefore, the surfaces with the stripe and two circular domains can be distinguished by the minor axis $D_{2}$.

**Figure 4:**
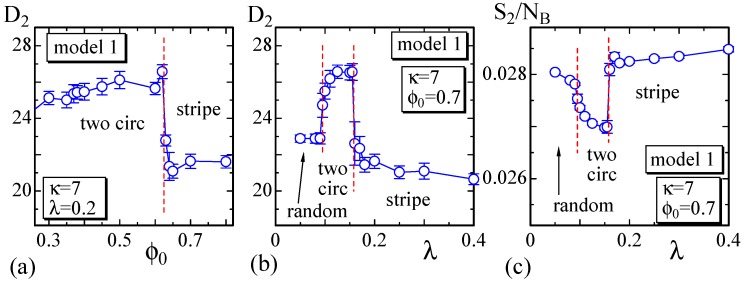
(**a**) The size $D_{2}$ vs. $\phi_{0}$ at λ=0.2; (**b**) $D_{2}$ vs. *λ* at $\phi_{0}=0.7$; and (**c**) the bending energy $S_{2}/N_{B}$ vs. *λ* at ϕ=0.7. These are calculated on the dashed horizontal and vertical lines in Figure 3. The minor axis $D_{2}$ and the bending energy $S_{2}/N_{B}$ change almost discontinuously and smoothly at the phase boundaries, which are denoted by the vertical dashed lines.

**Figure 5:**
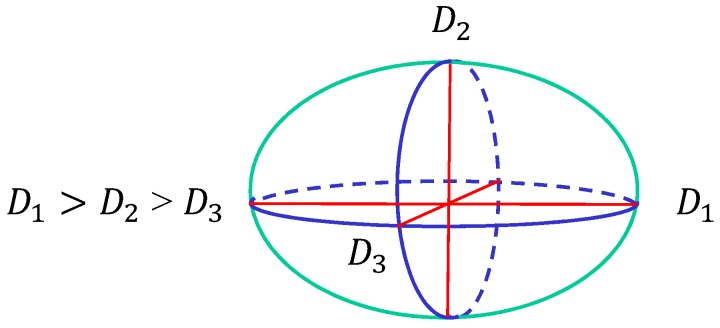
The surface size is characterized by three diameters $D_{1}$, $D_{2}$ and $D_{3}$, where $D_{1}>D_{2}>D_{3}$. The three axes are perpendicular to each other.

We plot $D_{2}$ vs. $\phi_{0}$ in Figure 4a, where λ=0.2. As shown, $D_{2}$ discontinuously changes against $\phi_{0}$ at the phase boundary between the two circular and stripe domains. From the plot of $D_{2}$ vs. *λ* in Figure 4b, we also observe that $D_{2}$ discontinuously changes at the same phase boundary. The bending energy $S_{2}/N_{B}$ in Figure 4c also discontinuously changes at this boundary, and this result indicates that this morphological change is considered as a first-order transition. However, note that the change of the morphology at this phase boundary is relatively smooth. In fact, one circular domain surface, which is not shown as a snapshot in Figure 3, can be observed at the boundary. This implies that the stripe domain surface and one circular domain surface have the same bending energy, or in other words, the bending energy is degenerate. Additionally, note that the phase boundary between the two circular and random domains appears to be continuous. This means that the shape of the two circular domain surfaces continuously changes to the random domain surface. At this phase boundary, the surface shape continuously changes from pancake to sphere.

### 4.2. Model 2

**Figure 6:**
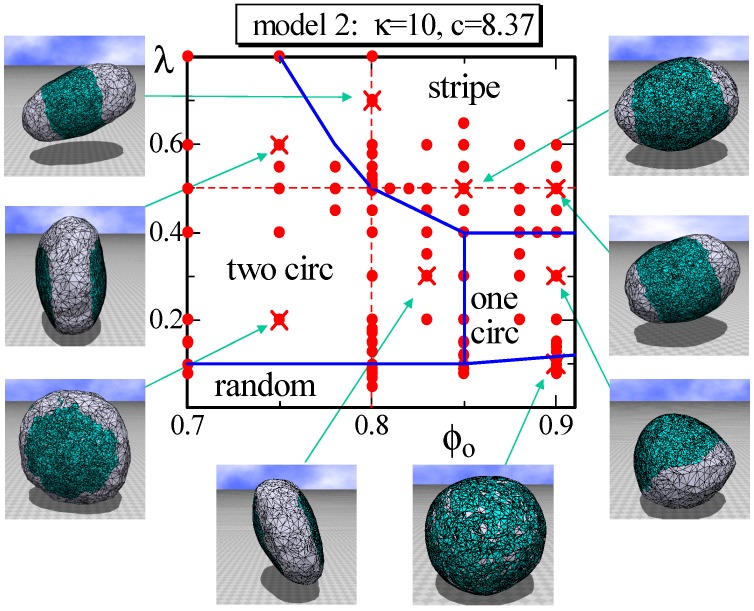
A phase diagram of Model 2 on the $\lambda -\phi_{o}$ plane at κ=10 and c=8.37 and the snapshots of surfaces obtained at the points indicated by the symbol (×). The solid lines on the phase diagram denote the phase boundaries, and the dashed lines denote the positions for the simulations for Figure 7a–c. The solid circles (•) denote the data points of the simulations for the phase boundaries.

In Model 2, not only $\kappa_{ij}$, but also $\gamma_{ij}$ depend on the domain (or the domain boundary) whether it is $L_{o}$ or $L_{d}$. For this reason, the area of the triangles in the $L_{o}$ domain becomes considerably smaller than that in the $L_{d}$ domain. Therefore, the fraction $\phi_{0}$ does not reflect the area fraction of $L_{o}$ in this case. In fact, it is easy to see that the area fraction of $L_{o}$ in the snapshots at $\phi_{0}=0.9$ in Figure 6 is much smaller than 90%. Nevertheless, the phase diagram on the $\lambda -\phi_{0}$ plane in Figure 6 appears almost the same as that in Figure 3. The only difference between the two phase diagrams is the appearance of one circular domain phase, denoted by “one circ” in Figure 6. This one circular phase is stable, where “stable” means that the surface domain remains unchanged against a small variation of the parameters inside the phase boundary. This is in sharp contrast to the one circular domain surfaces observed at the region close to the boundary between the two circular and stripe domains because these one circular surfaces are very sensitive to the parameter variation and, hence, “unstable”. The shape of the one circular surface in the one circular region is almost spherical, such as the one shown in Figure 6, and this result is in contrast to the result in [4], where the one circular phase is separated into two phases: the prolate and oblate phases. One possible reason for why only a spherical surface appears in the one circular domain in Figure 6 is because the $L_{o}$ domain is hardly bent due to the high ratio $\kappa_{ij}\left(L_{o},L_{o}\right)/\kappa_{ij}\left(L_{d},L_{d}\right)=4.24$, which is slightly larger than the one $1<\kappa_{ij}\left(L_{o},L_{o}\right)/\kappa_{ij}\left(L_{d},L_{d}\right)<3$ assumed in [4]. The parameters assumed on this plane are κ=10 and c=8.37, which are listed in Table 2.

The simulations are also performed on the $\lambda -\phi_{0}$ planes for larger *κ*, such as κ=15 and κ=20, and with c=8.37. The phase diagrams obtained in these simulations are (not shown) relatively close to those shown in Figure 6; however, surfaces with three or four circular domains appear in the lower *λ* region in the two circular domain phase. The bending energy $\kappa S_{2}$ of the three or four domains is lower than that of the two circular domain; moreover, the aggregation energy $\lambda S_{0}$ of these multi-circular domains is larger than that of the two circular domain. These are the reasons for the appearance of the three or four domains only in the relatively small *λ* region in the simulations with relatively large *κ*.

**Figure 7:**
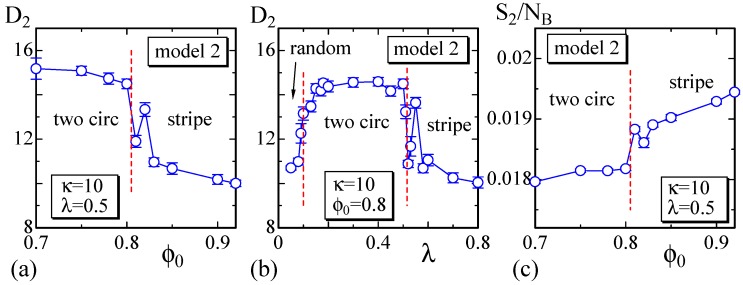
(**a**) The size $D_{2}$ vs. $\phi_{0}$ at λ=0.5; (**b**) $D_{2}$ vs. *λ* at $\phi_{0}=0.8$; and (**c**) the bending energy $S_{2}/N_{B}$ vs. $\phi_{0}$ at λ=0.5. These are calculated on the dashed horizontal and vertical lines in Figure 6. The size of the surface changes almost discontinuously and smoothly at the phase boundaries, which are denoted by the dashed lines.

To observe the variation of the surface size at the phase boundaries, we calculate the size $D_{2}$ on the dashed lines in Figure 6 and plot them in Figure 7a,b. We determine that $D_{2}$ discontinuously changes against $\phi_{0}$ and *λ* at the phase boundaries, similar to that in Model 1 shown in Figure 4a,b. Moreover, the phase boundary is also not as clear because of the same reason as that for Model 1. In fact, the surface shape at the phase boundary between the two circular and stripe domains is not always stable in Model 2, similar to that in Model 1. Figure 7c also shows that $S_{2}/N_{B}$ discontinuously changes; however, the gap is very small, and these two phases are hence separated by a weak first-order transition. The phase boundary between the two circular and the random domains is also expected to be continuous in Model 2. The boundaries of one circular to two circular and one circular to stripe are also not as clear, and the boundary of one circular to random is continuous.

**Figure 8:**
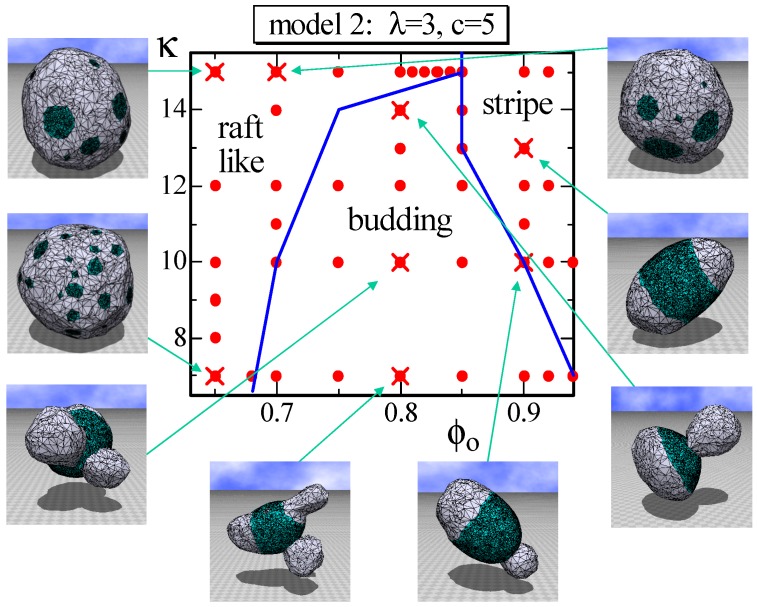
A phase diagram of Model 2 on the $\kappa -\phi_{o}$ plane at λ=3 and c=5 and the snapshots of surfaces obtained at the points indicated by the symbol (×). The solid lines on the phase diagram denote the phase boundaries. The solid circles (•) denote the data points of the simulations for the phase boundaries.

Another difference between Model 1 and Model 2, other than the appearance of the stable one circular domain, is the raft-like domain and the budding domain. More precisely, the budding domain can also be seen in Model 1; however, it is more clear in Model 2. The phase diagram of Model 2 on the $\kappa -\phi_{0}$ plane is drawn in Figure 8. The parameter *λ* is fixed to λ=3, which is relatively large compared to the previous one assumed in the simulations for Figure 3 and Figure 6. Consequently, the energy $\lambda S_{0}$, which is the line tension energy, at the phase boundary between $L_{0}$ and $L_{d}$ becomes large in the region where *κ* is relatively small. This is the reason why the budding domain appears on this $\kappa -\phi_{0}$ plane in Figure 8. Note that the budding domain in some of the budding surfaces goes inside the surface, and some of them self-intersect because no self-avoiding interaction is assumed. Figure 8 also shows that the raft-like domain is stable in the relatively large *κ* region, where the surface hardly deforms. The reason why the raft domains, which are multi-circular domains, appear only at the region of small $\phi_{0}$ is because the multi-circular domains are more energetically favorable than the stripe domain. Indeed, the effective bending rigidity $\kappa \kappa_{ij}$ and, hence, $\kappa S_{2}$ become very large on the large connected $L_{o}$ domain, such as the stripe domain, where the line tension energy $\lambda S_{0}$ is relatively small. Note that $S_{0}$ has a non-zero positive value only on the boundary bonds between $L_{o}$ and $L_{d}$, while $S_{2}$ has a non-zero value on all of the bonds. Moreover, note that the boundary length between $L_{o}$ and $L_{d}$ becomes longer (shorter) if the total number of circular domains increases (decreases), whereas the areas of $L_{o}$ and $L_{d}$ remain constant and are independent of the total number of $L_{o}$ domains.

**Figure 9:**
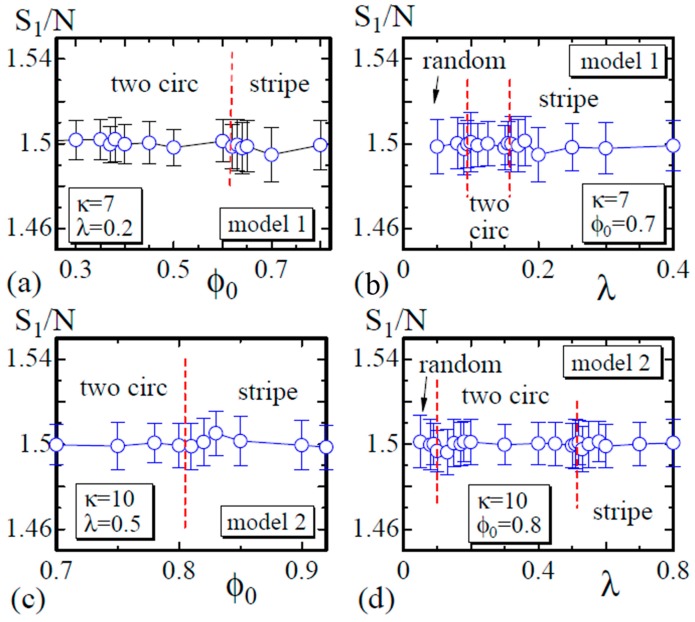
(**a**) The Gaussian energy $S_{1}/N$ vs. $\phi_{0}$ at λ=0.2; (**b**) $S_{1}/N$ vs. *λ* at $\phi_{0}=0.7$ for Model 1; (**c**) $S_{1}/N$ vs. $\phi_{0}$ at λ=0.5; and (**d**) $S_{1}/N$ vs. *λ* at $\phi_{0}=0.8$ for Model 2. The data in (**a**) and (**b**) ((**c**) and (**d**)) are obtained on the dashed lines in Figure 3 (Figure 6).

Finally, we show that $S_{1}/N$ satisfies the relation $S_{1}/N=1.5$, which is expected by the scale invariance of *Z* in Equation (6) [29]. As described in Section 2, the bond length is expected to be well defined in the sense that the mean bond length is constant on the surface, although this constant varies depending on the domains or the domain boundary to which the bond belongs. The data in Figure 9a,b are obtained on the dashed lines in Figure 3, and those in Figure 9c,d are obtained on the lines in Figure 6. These data shown in Figure 9 indicate that the simulations including the energy discretization are successful.

## 5. Summary and Conclusions

We have studied the phase separation of the three-component membrane with DPPC, DOPC and cholesterol using a Finsler geometry (FG) surface model. The FG model is obtained from the Helfrich–Polyakov (HP) model for membranes by replacing the surface metric with a general one $g_{ab}\neq \delta_{ab}$, which can be called the Finsler metric. In other words, we have extended the HP model to explain the morphological changes of the three-component membranes in the context of FG modeling. This new model includes a new degree of freedom *σ*, which represents the liquid-ordered ($L_{o}$) and liquid-disordered ($L_{d}$) domains. The results obtained from Monte Carlo (MC) simulations are consistent with the experimental results that have been reported in the literature. We confirm the phase separation of the $L_{o}$ and $L_{d}$ domains on the surface and that the surface shows a variety of morphologies, such as the two circular domain, the stripe domain, the raft domain and the budding domain.

The line tension energy, which has been used for understanding the morphological changes, simply corresponds to the aggregation energy term $\lambda S_{0}$ in our model. Indeed, the value of $S_{0}$ is only the total number of bonds on the boundary between $L_{o}$ and $L_{d}$ in our new model. Moreover, the fact that $\lambda S_{0}$ is simply the line tension energy implies that the line tension originates from the interaction between the domains because the interaction between the variables *σ* in $S_{0}$ describes the interaction between the domains. This interaction is closely connected to the property of the new model that the surface strength, such as the surface tension and the bending rigidity, is dependent on the bond position on the surface. This property arises from the interaction between *σ* and r introduced via the Finsler metric.

## Figures and Tables

**Figure A2:**
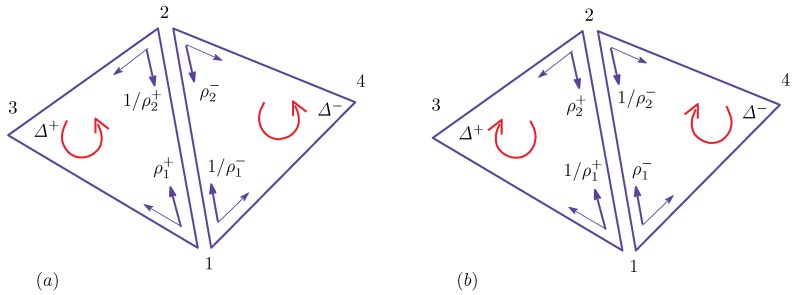
Local coordinate origins of the triangles $\Delta^{+}$ and $\Delta^{-}$ for $S_{1}\left(\ell_{12}\right)$ and the elements of $\gamma_{12}$ and $\gamma_{21}$ of the configurations of (**a**) the original and (**b**) the inside out (inside view).

**Table 1:** The Gaussian bond potential $S_{1}$ and the parameters $\gamma_{ij}$ and $\kappa_{ij}$ assumed in Model 1 and Model 2. (see Equation (A9) in Appendix A.1)

	$S_{1}$	$\gamma_{ij}$	$\kappa_{ij}$
Model 1	$S_{1}=\sum_{ij}\ell_{ij}^{2}$	1	$(c_{i}+c_{j})/4$
Model 2	$S_{1}=\sum_{ij}\gamma_{ij}\ell_{ij}^{2}$	$(c_{i}+c_{j})/4$	$(c_{i}+c_{j})/4$

**Table 2:** The input parameters *λ*, *κ*, *c* and $\phi_{0}$ for the simulations.

	*λ*	*κ*	*c*	$\phi_{0}$
Model 1	0.03≤λ≤0.5	7	5	$0.3\leq \phi_{0}\leq 0.8$
Model 2	0.03≤λ≤0.8	10	8.37	$0.7\leq \phi_{0}\leq 0.9$
Model 2	3	7≤κ≤15	5	$0.65\leq \phi_{0}\leq 0.95$

**Table 3:** The input parameter *c* automatically defines the values of $\kappa_{ij}$ and $\gamma_{ij}$, where $\gamma_{ij}=1$ for Model 1 and $\gamma_{ij}=\kappa_{ij}$ for Model 2.

	*c*	$\kappa_{ij}\left(L_{o},L_{o}\right)$	$\kappa_{ij}\left(L_{o},L_{d}\right)$	$\kappa_{ij}\left(L_{d},L_{d}\right)$
Model 1	5	2.6	1.8	1
Model 2	8.37	4.24	2.62	1
Model 2	5	2.6	1.8	1

## Abbreviations

The following abbreviations are used in this manuscript:

MC: Monte Carlo
MCS: Monte Carlo sweep
$L_{o}$: Liquid-ordered
$L_{d}$: Liquid-disordered
DOPC: Dioleoylphosphatidylcholine
DPPC: Dipalmitoylphosphatidylcholine
HP: Helfrich and Polyakov
FG: Finsler geometry

## Appendix A Finsler Geometry Modeling

### Appendix A.1. Discrete Surface Model

To obtain the discrete model from the continuous surface model introduced in Section 2.1, we assume that the surface is triangulated in ${ℜ}^{3}$. The Hamiltonian is defined on the triangulated surfaces, which are composed of three simplexes, such as vertices, bonds and triangles. Thus, all physical quantities, including Hamiltonians and the metric function, are defined on these simplexes labeled by integers. For example, the vertex position $r_{i}$ is defined at the vertex *i*; the bond length $\ell_{ij}$ is defined on the bond ij; and the elements of $g_{ab}$ are defined on the triangle *Δ*. Note that the variable r is considered as a mapping from the parameter space *M* to ${ℜ}^{3}$.

We start with the discrete metric $g_{ab}$, such that:

(A1) $$g_{ab}=\left(\begin{array}{cc} 1/\rho & 0\\ 0 & \rho \end{array}\right),\;\rho >0,$$

where *ρ* is a function on a triangle $\Delta (\subset {ℜ}^{3})$ (see Figure A1a). More precisely, the elements of $g_{ab}$ are functions on the triangle $\Delta_{M}\left(\subset M\right)$, where *M* is the aforementioned two-dimensional space *M* (independent of ${ℜ}^{3}$). We assume that *M* is also triangulated by the triangles $\Delta_{M}$. On this $\Delta_{M}$, an orthogonal coordinate can be taken for any one of three vertices [14]. For this reason, the metric $g_{ab}$ can be diagonalizable. The inequality ρ>0 in Equation (A1) is necessary for the positivity of the bond length. This metric depends only on *x* and is independent of *y*, and hence, it simply corresponds to the Riemannian metric. Indeed, this metric in Equation (A1) comes from the most general one, such as $g_{ab}=\left(\begin{array}{cc} E & \;F\\ F & \;G \end{array}\right)$, with the functions of $E>0,\;G>0,\;EG-F^{2}>0$. By assuming F=0, we have $g_{ab}=\left(\begin{array}{cc} E & \;0\\ 0 & \;G \end{array}\right)=E\left(\begin{array}{cc} 1 & \;0\\ 0 & \;G/E \end{array}\right)≃\left(\begin{array}{cc} 1 & \;0\\ 0 & \;\rho^{2} \end{array}\right)≃\left(\begin{array}{cc} 1/\rho & \;0\\ 0 & \;\rho \end{array}\right)$, where $\rho^{2}=G/E$ and the symbol “≃” denotes conformally equivalent. Note that this expression of $g_{ab}$ depends on the local coordinates on *Δ*, and therefore, the expression of $g_{ab}$ implicitly depends on the vertex of *Δ* because the coordinate origin is located on one of the vertices of *Δ*. In the discrete models that have been studied thus far, the Euclidean metric $g_{ab}=\delta_{ab}$ (or the induced metric $g_{ab}=\partial_{a}r\cdot \partial_{b}r$) is always assumed as mentioned above, and it has been reported that the model for polymerized membranes undergoes a discontinuous or a continuous transition between the crumpled phase and the smooth phase [32,33,34,35].

Let the vertex $r_{1}$ of the central triangle *Δ* in Figure A1b be the local coordinate origin in this *Δ*. By replacing:

(A2) $$\begin{array}{c} \int \sqrt{g}d^{2}x\to \sum_{\Delta },\\ \frac{\partial r}{\partial x_{1}}\to r_{2}-r_{1},\;\frac{\partial r}{\partial x_{2}}\to r_{3}-r_{1},\\ \frac{\partial n}{\partial x_{1}}\to n_{0}-n_{2},\;\frac{\partial n}{\partial x_{2}}\to n_{0}-n_{1}, \end{array}$$

we have:

(A3) $$\begin{array}{c} S_{1}=\sum_{\Delta }S_{1}\left(\Delta \right)=\sum_{\Delta }\left(\rho \ell_{12}^{2}+\frac{1}{\rho }\ell_{13}^{2}\right),\\ S_{2}=\sum_{\Delta }S_{2}\left(\Delta \right)=\sum_{\Delta }\left[\rho \left(1-n_{0}\cdot n_{1}\right)+\frac{1}{\rho }\left(1-n_{0}\cdot n_{2}\right)\right], \end{array}$$

where $n_{i}\left(i=1,2,3\right)$ are the unit normal vectors shown in Figure A1b. The symbol $\ell_{ij}\left(=\ell_{ji}\right)$ is defined by $\ell_{ij}=\left|r_{j}-r_{i}\right|$. Note that the unit normal vector also represents the surface orientation; indeed, $n_{0}$ is defined by $n_{0}={\vec{\ell }}_{12}\times {\vec{\ell }}_{13}/\left|{\vec{\ell }}_{12}\times {\vec{\ell }}_{13}\right|$, for example.

We have three possible coordinate origins in the triangles. For this reason, $S_{1}$ and $S_{2}$ can be symmetrized by including the terms that are cyclic permutations, such as 1→2, 2→3, 3→1 for $\ell_{ij}$, $n_{i}$ and $\rho_{i}$. Summing over all possible terms and multiplying by a factor of 1/3, we obtain:

(A4) $$\begin{array}{cc} S_{1}= & \frac{1}{3}\sum_{\Delta }\left[\left(\rho_{1}+\frac{1}{\rho_{2}}\right)\ell_{12}^{2}+\left(\rho_{2}+\frac{1}{\rho_{3}}\right)\ell_{23}^{2}+\left(\rho_{3}+\frac{1}{\rho_{1}}\right)\ell_{31}^{2}\right],\\ S_{2}= & \frac{1}{3}\sum_{\Delta }\left[\left(\rho_{1}+\frac{1}{\rho_{2}}\right)\left(1-n_{0}\cdot n_{1}\right)+\left(\rho_{2}+\frac{1}{\rho_{3}}\right)\left(1-n_{0}\cdot n_{3}\right)\right.\\ & +\left.\left(\rho_{3}+\frac{1}{\rho_{1}}\right)\left(1-n_{0}\cdot n_{2}\right)\right], \end{array}$$

where $\rho_{i}\left(i=1,2,3\right)$ are defined on the triangle *Δ*. The reason for why these three different functions $\rho_{i}$ are included is because the expression for $g_{ab}$ generally depends on the local coordinate as mentioned above. More precisely, $\rho_{i}$ is the element of $g_{ab}$ on *Δ* where the coordinate origin is located at vertex *i*. For arbitrary $g_{ab}$, we always have the metric of the form in Equation (A1) by the same procedure as described above.

Here, we further simplify the model by assuming that:

(A5) $$\begin{array}{c} \rho_{1}=\rho_{2}=\rho_{3}\left(=\rho_{\Delta }\right). \end{array}$$

Thus, we have the expressions:

(A6) $$\begin{array}{c} S_{1}=\frac{1}{3}\sum_{\Delta }\left(\rho_{\Delta }+\frac{1}{\rho_{\Delta }}\right)\left(\ell_{12}^{2}+\ell_{23}^{2}+\ell_{31}^{2}\right),\\ S_{2}=\frac{1}{3}\sum_{\Delta }\left(\rho_{\Delta }+\frac{1}{\rho_{\Delta }}\right)\left(1-n_{0}\cdot n_{1}+1-n_{0}\cdot n_{2}+1-n_{0}\cdot n_{3}\right). \end{array}$$

Replacing the sum over triangles $\sum_{\Delta }$ with the sum over bonds $\sum_{ij}$, we obtain:

(A7) $$\begin{array}{c} S_{1}=\frac{1}{3}\sum_{ij}\left(\rho_{i}+\frac{1}{\rho_{i}}+\rho_{j}+\frac{1}{\rho_{j}}\right)\ell_{ij}^{2},\\ S_{2}=\frac{1}{3}\sum_{ij}\left(\rho_{i}+\frac{1}{\rho_{i}}+\rho_{j}+\frac{1}{\rho_{j}}\right)\left(1-n_{i}\cdot n_{j}\right). \end{array}$$

Note that $S_{1}$ and $S_{2}$ defined by the sum over triangles $\sum_{\Delta }$ in Equation (A6) are exactly the same as those defined by the sum over bonds $\sum_{ij}$ in Equation (A7), and the difference is only in their expressions. Additionally, note that the suffixes i,j of $\ell_{ij}$ in Equation (A7) denote the bond ij, whereas those of $\rho_{i}$ and $\rho_{j}$ denote the two neighboring triangles *i* and *j* of the bond ij. Thus, we finally have:

(A8) $$\begin{array}{c} S\left(r,\sigma \right)=S_{1}+\kappa S_{2},\\ S_{1}=\sum_{ij}\gamma_{ij}\ell_{ij}^{2},\;S_{2}=\sum_{ij}\kappa_{ij}\left(1-n_{i}\cdot n_{j}\right), \end{array}$$

with:

(A9) $$\gamma_{ij}=\kappa_{ij}=\frac{c_{i}+c_{j}}{4},\;c_{i}=\rho_{i}+\frac{1}{\rho_{i}},$$

where the irrelevant numerical factor 1/3 is replaced by 1/4 in the final expressions for $S_{1}$ and $S_{2}$.

Note that $\gamma_{ij}$ ($\kappa \kappa_{ij}$) can be called the effective surface tension (effective bending rigidity) on the bond between vertices *i* and *j*. It must be emphasized that the quantities $\gamma_{ij}$ and $\kappa_{ij}$ are independent of the bond direction, or in other words, these are symmetric under the exchange of *i* and *j*, and for this reason, $\gamma_{ij}$ and $\kappa_{ij}$ are considered as the quantities defined on the bond ij. Indeed, from the expression given in Equation (A9), we have:

(A10) $$\gamma_{ij}=\gamma_{ji},\;\kappa_{ij}=\kappa_{ji}.$$

Therefore, the physical quantities $\gamma_{ij}\ell_{ij}^{2}$ in $S_{1}$ and $\kappa_{ij}\left(1-n_{i}\cdot n_{j}\right)$ in $S_{2}$ of Equation (A8) are well defined in the sense that these quantities are symmetric under the exchange of ij. The reason why we need this symmetry in the physical quantities $\gamma_{ij}\ell_{ij}^{2}$ and $\kappa_{ij}\left(1-n_{i}\cdot n_{j}\right)$ is because these quantities correspond to the energies for the expansion and bending of the surface at the bond ij, and these energies are independent of the bond direction, such as the one from *i* to *j* or the reverse. Thus, the symmetry property in Equation (A10) allows us to call $\gamma_{ij}$ and $\kappa \kappa_{ij}$ the effective surface tension and the effective bending rigidity on the bond ij, respectively. However, as we will show in the next subsection, $\gamma_{ij}$ and $\kappa_{ij}$ are not symmetric in general (see Figure A1c). Moreover, note that this problem of whether $\gamma_{ij}$ and $\kappa_{ij}$ are symmetric arises only when $\gamma_{ij}$ and $\kappa_{ij}$ depend on the functions $\rho_{i}$ and $\rho_{j}$ on the two neighboring triangles *i* and *j*. This is in sharp contrast to the case where $\gamma_{ij}$ and $\kappa_{ij}$ depend only on the quantity defined on the vertices [10], where $\gamma_{ij}$ and $\kappa_{ij}$ are always symmetric. This exchange symmetry/asymmetry reflects the orientation symmetry/asymmetry, which will be discussed in the next subsection.

### Appendix A.2. Finsler Geometry Model

In this subsection, we show that the discrete surface models constructed above are well defined only in the context of Finsler geometry modeling [10]. For this purpose, we should first remind ourselves of the fact that the symmetry properties in Equation (A10) can be observed in the model only under the condition of Equation (A5). This symmetry is not present in the model of Equation (A4). To show the breakdown of the symmetry of Equation (A10) in the model of Equation (A4) in more detail, we replace the sum over triangles $\sum_{\Delta }$ of $S_{1}$ and $S_{2}$ in Equation (A4) with the sum over bonds $\sum_{ij}$ before the condition of Equation (A5) is imposed. In this new expression of $S_{1}$, which is expressed by the sum over bonds $\sum_{ij}$, the Gaussian bond potential for the bond 12, which is shared by the triangles $\Delta^{+}$ and $\Delta^{-}$ as in Figure A2a, for example, is given by:

(A11) $$S_{1}\left(\ell_{12}\right)=\left(1/3\right)\left(\rho_{1}^{+}+\frac{1}{\rho_{2}^{+}}+\rho_{2}^{-}+\frac{1}{\rho_{1}^{-}}\right)\ell_{12}^{2}∼\gamma_{12}\ell_{12}^{2}.$$

In this expression, the former half $S_{1}^{+}\left(\ell_{12}\right)=\left(1/3\right)\left(\rho_{1}^{+}+1/\rho_{2}^{+}\right)\ell_{12}^{2}$ is the contribution from $\Delta^{+}$, and the latter half $S_{1}^{-}\left(\ell_{12}\right)=\left(1/3\right)\left(\rho_{2}^{-}+1/\rho_{1}^{-}\right)\ell_{12}^{2}$ is the contribution from $\Delta^{-}$. However, it is clear that $\gamma_{12}$, and hence, $S_{1}\left(\ell_{12}\right)$ in Equation (A11) are not symmetric under the change of surface orientation. In fact, we have:

(A12) $${\bar{S}}_{1}\left(\ell_{12}\right)=\left(1/3\right)\left(\rho_{2}^{+}+\frac{1}{\rho_{1}^{+}}+\rho_{1}^{-}+\frac{1}{\rho_{2}^{-}}\right)\ell_{12}^{2}∼\gamma_{21}\ell_{12}^{2}$$

for the opposite orientation (see Figure A2b). In Equation (A12), we write the coefficient of $\ell_{12}^{2}$ by $\gamma_{21}$ because it is obtained from $\gamma_{12}$ in Equation (A11) by exchanging the suffixes 1 and 2. It is also easy to show that $\gamma_{12}\neq \gamma_{21}$, and hence, $S_{1}\left(\ell_{12}\right)$ are not always identical to ${\bar{S}}_{1}\left(\ell_{12}\right)$ in general.

Thus, we find that the asymmetry $\gamma_{12}\neq \gamma_{21}$ means that $S_{1}\left(\ell_{12}\right)$ is not invariant under the orientation exchange. From this, we can see that the Gaussian bond potential energy (and also the bending energy) of the bond 12 of one surface configuration differs from that of the opposite orientation configuration. However, we have no reason for the difference in $S_{1}$ for two surfaces with different orientations. Thus, the model defined by Equation (A4), which is orientation asymmetric, is ill defined in the context of conventional surface modeling.

Moreover, we have to remark that the model defined by Equation (A8), which is orientation symmetric, is also ill defined. The reason for this ill definedness is that the bond length squares calculated with $\rho^{+}$ in $\Delta^{+}$ is not always identical to the one calculated with $\rho^{-}$ in $\Delta^{-}$ in Figure A2a, where $\rho_{1}^{\pm }=\rho_{2}^{\pm }$ in the model of Equation (A8). Indeed, the metric on $\Delta^{+}$ is given by $g_{ab}=\left(\begin{array}{cc} 1/\rho^{+} & \;0\\ 0 & \;\rho^{+} \end{array}\right)$, where the coordinate origin is at the vertex 1. Then, we have the bond length squares $\left(1/\rho^{+}\right)\ell_{12}^{2}$ for the bond 12 with respect to the metric $g_{ab}$, and changing the vertex origin from 1 to 2, we also have $\rho^{+}\ell_{12}^{2}$. Thus, summing over these two expressions without the coefficient 1/2, we have $\left(1/\rho^{+}+\rho^{+}\right)\ell_{12}^{2}$ for the bond length squares. Through the same procedure, we have $\left(1/\rho^{-}+\rho^{-}\right)\ell_{12}^{2}$ from $\Delta^{-}$. These two square lengths of the bond 12 must be the same. However, we have:

(A13) $$\frac{1}{\rho^{+}}+\rho^{+}\neq \frac{1}{\rho^{-}}+\rho^{-}$$

because $\rho^{+}\neq \rho^{-}$ in general. The edge length of triangles should uniquely be given as the basic requirement even in the discrete models. Therefore, in a model construction on the triangulated lattices, we always obtain an ill-defined discrete model if we start with an arbitrary Riemannian metric in which the elements are defined on the triangles. Note that the bond “length” used here is the length with respect to $g_{ab}$ on $\Delta^{\pm }$ and is different from the Euclidean bond length $\ell_{ij}$ (also note that $g_{ab}$ is simply a Riemannian metric at this stage).

However, these ill-defined models in Equations (A4) and (A8) become well defined in the context of Finsler geometry [10,11,12]. In this context, the bond length calculated with $g_{ab}$ on the triangle $\Delta^{+}$ can be considered as the direction-dependent length from 1 to 2, and the one calculated with $g_{ab}$ on $\Delta^{-}$ can be considered as the length from 2 to 1 (Figure A2a). Therefore, the inequality in Equation (A13) is satisfied in general. Moreover, the aforementioned quantities $S_{1}^{+}\left(\ell_{12}\right)$ and $S_{1}^{-}\left(\ell_{12}\right)$ in Equations (A11) and (A12) are meaningful because these quantities are also considered as direction dependent in the Finsler geometry context (Figure A1c).
